# Extracellular matrix and α_5_β_1_ integrin signaling control the maintenance of bone formation capacity by human adipose-derived stromal cells

**DOI:** 10.1038/srep44398

**Published:** 2017-03-14

**Authors:** Nunzia Di Maggio, Elisa Martella, Agne Frismantiene, Therese J. Resink, Simone Schreiner, Enrico Lucarelli, Claude Jaquiery, Dirk J. Schaefer, Ivan Martin, Arnaud Scherberich

**Affiliations:** 1 Laboratory of Tissue Engineering, Department of Surgery, University Hospital of Basel and Department of Biomedicine, University of Basel, Switzerland; 2 Osteoarticular Regeneration Laboratory, Istituto Ortopedico Rizzoli, Bologna, Italy; 3 Department of Biomedical and Neuromotor Sciences (DIBINEM), University of Bologna, Italy; 4 Laboratory of Signal Transduction, Department of Biomedicine, University Hospital Basel, Basel, Switzerland; 5 Clinic for Oral and Maxillofacial Surgery, University Hospital of Basel, Basel, Switzerland; 6 Department of Plastic, Reconstructive, Aesthetic and Hand Surgery, University Hospital of Basel, Switzerland

## Abstract

Stromal vascular fraction (SVF) cells of human adipose tissue have the capacity to generate osteogenic grafts with intrinsic vasculogenic properties. However, adipose-derived stromal/stem cells (ASC), even after minimal monolayer expansion, display poor osteogenic capacity *in vivo*. We investigated whether ASC bone-forming capacity may be maintained by culture within a self-produced extracellular matrix (ECM) that recapitulates the native environment. SVF cells expanded without passaging up to 28 days (Unpass-ASC) deposited a fibronectin-rich extracellular matrix and displayed greater clonogenicity and differentiation potential *in vitro* compared to ASC expanded only for 6 days (P0-ASC) or for 28 days with regular passaging (Pass-ASC). When implanted subcutaneously, Unpass-ASC produced bone tissue similarly to SVF cells, in contrast to P0- and Pass-ASC, which mainly formed fibrous tissue. Interestingly, clonogenic progenitors from native SVF and Unpass-ASC expressed low levels of the fibronectin receptor α_5_ integrin (CD49e), which was instead upregulated in P0- and Pass-ASC. Mechanistically, induced activation of α_5_β_1_ integrin in Unpass-ASC led to a significant loss of bone formation *in vivo*. This study shows that ECM and regulation of α_5_β_1_-integrin signaling preserve ASC progenitor properties, including bone tissue-forming capacity, during *in vitro* expansion.

Bone tissue engineering aims to generate biological substitutes to replace traumatic, neoplastic or degenerative bone loss and to correct related misalignment^1^. A typical approach comprises the harvesting of osteogenic progenitor cells from suitable tissues and their expansion *in vitro*, followed by loading into biocompatible scaffolding materials within which the cells can differentiate and generate bone tissue. Among possible sources, the freshly-isolated stromal vascular fraction (SVF) of human adipose tissue contains not only multipotent mesenchymal progenitors, referred to as adipose-derived stromal/stem cells (ASC), but also cells with vasculogenic phenotype^2^. Freshly-isolated, native SVF cells can generate bone and functional blood vessels *in vivo* if suitably induced by three-dimensional (3D) culture under perfusion flow^3^ or osteoinductive trigger^4^. However, even minimally monolayer-expanded SVF cells lose their osteogenic capacity^3^, unless they are either pre-differentiated into osteoblasts^5^,^6^ or chondrocytes^7^,^8^ *in vitro*, or exposed to bone morphogenetic protein-2 (BMP-2)^9^. These data suggest that SVF cells cannot maintain their osteoprogenitor properties during monolayer expansion on tissue culture plastic. A likely hypothesis is that monolayer culture does not fully mirror the physiological microenvironment of the stem cell niche, which regulates ASC differentiation potential and *in vivo* functionality^3^,^10^,^11^,^12^,^13^.

Different approaches have been attempted to provide appropriate signals during monolayer culture, in order to maintain osteogenic differentiation potential, including the use of different growth factors or expansion on different substrates or architectures. For example, inclusion of fibroblast growth factor -2 (FGF-2) was shown not only to stimulate proliferation, but also to select a subset of bone marrow mesenchymal progenitors (BMSC) with early progenitor characteristics^14^,^15^. Clonal growth and differentiation capacity of BMSC towards mesenchymal lineages were better preserved when mesenchymal progenitors were cultured on 3D scaffolds under direct perfusion^16^, underlining the importance of culture-substrate composition and architecture, as well as cell-cell and cell-extracellular matrix (ECM) interactions. The ECM is a fundamental component of specialized niches and might provide both architectural elements and non-structural molecules to influence the mechanical properties of the tissue and the differentiation capacity of progenitor cells^17^. Modulation of the differentiation potential of mesenchymal cells by ECM was previously demonstrated *ex vivo*. Compared to culture on plastic, adult BMSC cultured on ECM produced by fetal mesenchymal stem cells exhibited enhanced *in vitro* expansion and differentiation capacity^18^. Similarly, ASC expanded on an ECM formed by monolayer-grown BMSC maintained a higher *in vivo* osteogenic potential than ASC expanded directly on tissue culture plastic^19^. However, the precise mechanisms whereby niche elements such as cell-cell specific interactions or ECM elements can influence the differentiation potential of mesenchymal cells remain mostly unknown.

In the present study, we investigated whether sustained *in vitro* culture of SVF cells without passaging might allow the deposition of a self-produced ECM which could provide signals preserving ASC native progenitor properties.

## Results

### Unpass-ASC display higher clonogenicity and increased *in vitro* differentiation potential as compared to Pass-ASC

**Figure 1:**
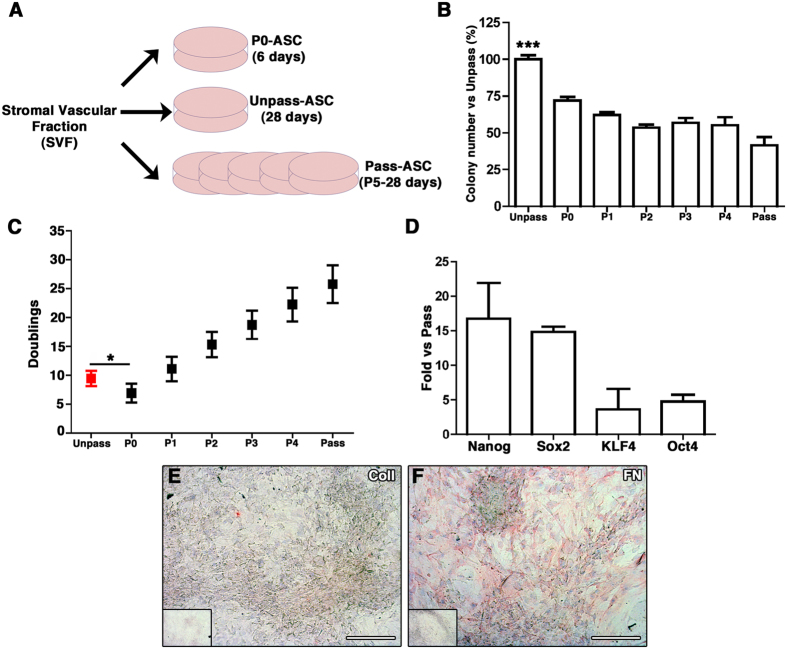
(**A**) Schematic representation of the protocol used to generate P0, Unpass- and Pass-ASC from SVF cells; (**B**) Number of colonies (expressed as percentage of colonies in the Unpass-ASC condition) in Unpass-ASC and from P0 (P0-ASC) to P5 (Pass-ASC) (***p < 0.001 for Unpass-ASC vs all other groups, ANOVA with Bonferroni multiple comparisons test); (**C**) Number of population doublings performed by Unpass- and Pass-ASC from P0 to P5 (*p < 0.05, ANOVA with Bonferroni multiple comparisons test); (**D**) Quantification of mRNA expression for Nanog, Sox2, KLF4 and Oct4 in Unpass-ASC expressed as fold vs. Pass-ASC. (**E,F**) Immunohistochemical analysis for Collagen type I (**E**) and fibronectin (**F**) of Unpass-ASC-produced ECM; Size bar = 100 μm.

Unpass-, P0 and Pass-ASC were generated from SVF cells (Fig. 1A) plated on tissue plastic dishes and grown either for 6 or 28 days without passaging at cell confluence (P0- and Unpass-ASC respectively) or, when reaching 80% of confluence, detached and reseeded into new dishes (Pass-ASC) for the same time (28 days). At the end of the culture period, Unpass-ASC contained a significantly higher number of clonogenic cells as compared to Pass-ASC (100 ± 6.7% and 41.5 ± 14.2% of the colonies present in the Unpass condition respectively, Fig. 1B, n = 6, p < 0.001). It was previously shown that for bone marrow mesenchymal progenitors, clonogenicity is drastically reduced by monolayer expansion^20^. Therefore, we determined whether the increased clonogenicity in Unpass-ASC was associated with a difference in the number of doublings undergone by the two populations. We assessed the number of population doublings in Unpass-ASC and at every passage (n = 11, Fig. 1C). Unpass-ASC performed 9.5 ± 1.3 population doublings, which was significantly higher than the ones undergone by P0-ASC (6.5 ± 1.6, p < 0.05). Interestingly, despite a higher number of doublings performed, Unpass-ASC still displayed a significantly increased clonogenitcity than P0-ASC (100 ± 6.7% and 71.9 ± 5.9% of colonies present in the Unpass condition, Fig. 1B, p < 0.001). Pass-ASC (P5) proliferated significantly more than both Unpass- and P0-ASC (25.8 ± 3.3 doublings, n = 5, p < 0.05) and also gave rise to less colonies, as expected. Furthermore, Unpass-ASC expressed higher levels of genes involved in the maintenance of multipotency, as shown by gene expression analysis for Oct-4, Sox-2, Nanog and KLF4 (Fig. 1D). Characterization of the composition of the ECM deposited after 28 days of culture showed that Unpass-ASC produced a matrix that stained negatively for collagen type I and was strongly positive for Fibronectin (FN) (Fig. 1E and F respectively).

**Figure 2:**
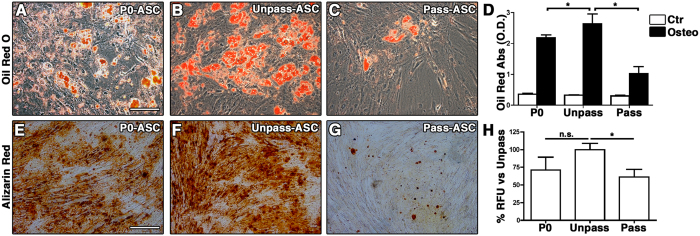
(**A–C**) Representative microscopic fields of Oil Red O staining of P0, Unpass- and Pass-ASC (Size bar = 100 μm); (**D**) Quantification of Oil Red O staining of P0, Unpass- and Pass-ASC cultured in control (white bar) or adipogenic medium (black bar); (**E–G**) Representative microscopic fields of alizarin Red staining of P0, Unpass- and Pass-ASC. Size bar = 100 μm; (**H**) Quantification of hydroxyapatite deposits in the P0, Unpass- and Pass-ASC conditions (results are expressed as percentage of the Unpass-ASC condition). *P < 0.05, n.s. = not significantly different (ANOVA with Bonferroni multiple comparisons test).

To compare their *in vitro* differentiation potential, Unpass-ASC, P0-ASC and Pass-ASC were differentiated towards the adipogenic and the osteogenic lineages (n = 3, Fig. 2). Unpass-ASC (Fig. 2A) gave rise to more adipocytes than P0- and Pass-ASC in culture (Fig. 2B and C respectively), as determined by quantification of Oil Red-O staining (Fig. 2D, p < 0.05). Following osteogenic stimulation, Unpass-ASC (Fig. 2E) produced more calcium deposits than Pass-ASC (Fig. 2G) as shown by alizarin red staining and also significantly higher amounts of hydroxyapatite (Fig. 2H, p < 0.05). No significant difference was observed between Unpass-ASC and P0-ASC (Fig. 2F,H, p = n.s.).

### Unpass-ASC form bone tissue *in vivo* similarly to SVF cells

**Figure 3:**
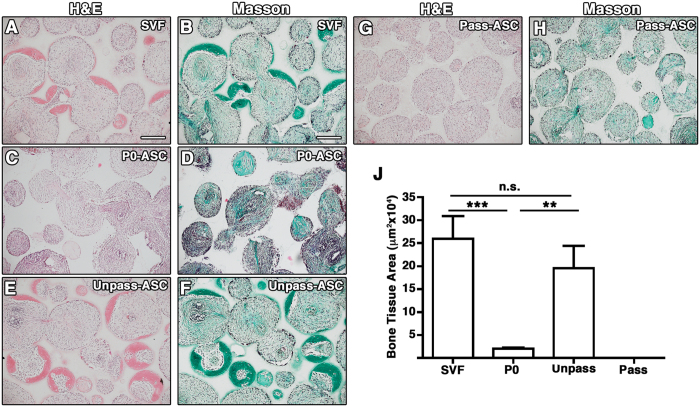
Representative microscopy fields of histological sections of constructs loaded with SVF cells (**A,B**), P0 (**C,D**), Unpass- (**E,F**) and Pass-ASC (**G,H**), stained with H&E (**A,C,E,G**) and Masson’s trichrome staining (**B,D,F,H**). Size bar = 200 μm; (**J**) Quantification of bone tissue formation area, **p < 0.01, ***p < 0.001, n.s. = not significantly different (Kruskal-Wallis with Dunn multiple comparisons test).

To confirm that Unpass-ASC display higher differentiation potential than P0- and Pass-ASC, their *in vivo* ectopic bone forming efficiency was tested and compared to SVF cells from the same donor (n = 3). Bone formation was evidenced by dense collagenous matrix embedding osteocytes formed within the pores of the ceramic scaffold (Fig. 3 and S1) in H&E (pink) and Masson’s trichrome (green) staining in the constructs loaded with SVF cells (Fig. 3A and B), Unpass- (Fig. 3E and F) and P0-ASC (Fig. 3C and D). Histomorphometric analysis showed no significant difference in the amount of ectopic bone formed by SVF cells and Unpass-ASC (Fig. 3J, 25.9 ± 4.9 × 10^4^ μm^2^ and 19.6 ± 4.8 × 10^4^ μm^2^ per histological section, respectively, p = n.s.). However, Unpass-ASC and SVF cells generated a 10-fold higher amount of bone tissue as compared to P0-ASC (2.0 ± 2.1 × 10^4^ μm^2^, n = 3, p < 0.01 and p < 0.001, respectively). No bone tissue formation was observed in the constructs containing Pass-ASC (Fig. 3G,H and J).

### Unpass-ASC and freshly isolated SVF cells have similar phenotype

We then addressed whether the superior differentiation ability of Unpass-ASC correlated with a specific phenotype. SVF cells, P0-, Unpass- and Pass-ASC were characterized for a complete set of antigens which included mesenchymal markers and various integrins (Table 1). All four populations were positive for CD73, CD90 and β_1_-integrin (CD29). In SVF cells and Unpass-ASC only a small population expressed the main collagen receptors α_1_-integrin (CD49a), α_2_-integrin (CD49b), α_3_-integrin (CD49c), α_4_-integrin (CD49d) and the vitronectin receptor CD41/61 (α_V_β_3_-integrin), which were instead homogenously expressed in P0- and Pass-ASC (>90%). Furthermore, all conditions expressed the FN receptor α_5_-integrin (CD49e). However, SVF cells and Unpass-ASC expressed low levels of CD49e, as compared to P0 and Pass-ASC. CD105 was expressed only by few cells in SVF and Unpass-ASC, while it was homogenously expressed by P0- and Pass-ASC. Moreover, the presence of a population of mature hematopoietic and endothelial cells could be observed in SVF cells, P0- and Unpass-ASC, but not in Pass-ASC.

**Figure 4:**
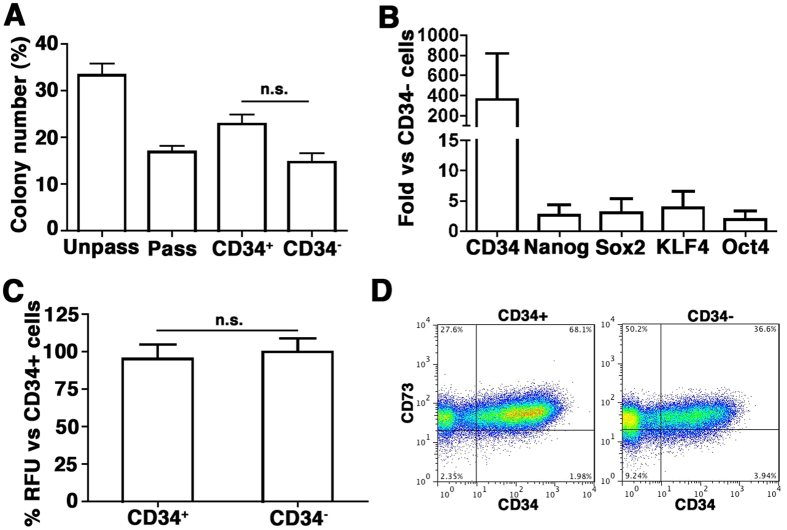
(**A**) Number of colonies, expressed as percentage of plated cells, for Unpass-, Pass-ASC, CD34^+^ and CD34^−^ sorted cells. n.s. = not significantly different (ANOVA with Bonferroni multiple comparisons test); (**B**) Quantification of mRNA expression for CD34, Nanog, Sox2, KLF4 and Oct4 in the CD34^+^ fraction of cells, expressed as fold vs CD34^−^ cells; (**C**) Quantification of hydroxyapatite deposits expressed as percentage of the CD34^+^ condition. n.s. = not significantly different (Mann-Whitney); (**D**) Representative cytofluorimetry plots for CD34 and CD73 of CD34^+^ and CD34^−^ sorted cells cultured as monolayer for 2 weeks after sorting.

Interestingly, ~30% of Unpass-ASC expressed CD34 (35.3 ± 14.7%, n = 3). Since ASC in native human adipose tissue express CD34^21^, we hypothesized that the increased clonogenic and differentiation potential of Unpass-ASC was due to the presence of the CD34^+^ subpopulation maintaining native progenitor properties. To test this hypothesis, Unpass-ASC were sorted according to CD34 expression (n = 3) and CD34^+^ and CD34^−^ cells were characterized for clonogenicity, gene expression and osteogenic differentiation potential *in vitro* (Fig. 4). CD34^+^ cells did not display higher clonogenicity as compared to CD34^−^ cells (Fig. 4A, p = n.s.). Furthermore, CD34^+^ and CD34^−^ populations did not differ significantly with respect to their expression of stem cell-associated genes (Fig. 4B, p = n.s.) or in the osteogenic differentiation potential *in vitro* (Fig. 4C, p = n.s.). Seeding on plastic tissue culture dishes after sorting also showed that the expression of CD34 in mesenchymal cells was modulated by cell confluence: 14 days after sorting, CD34 expression was upregulated in CD34^−^/CD73^+^ cells albeit to a lower extent than in CD34^+^/CD73^+^ cells (Fig. 4D).

### Unpass-ASC and freshly isolated SVF cells have similar adhesion properties to fibronectin

**Figure 5:**
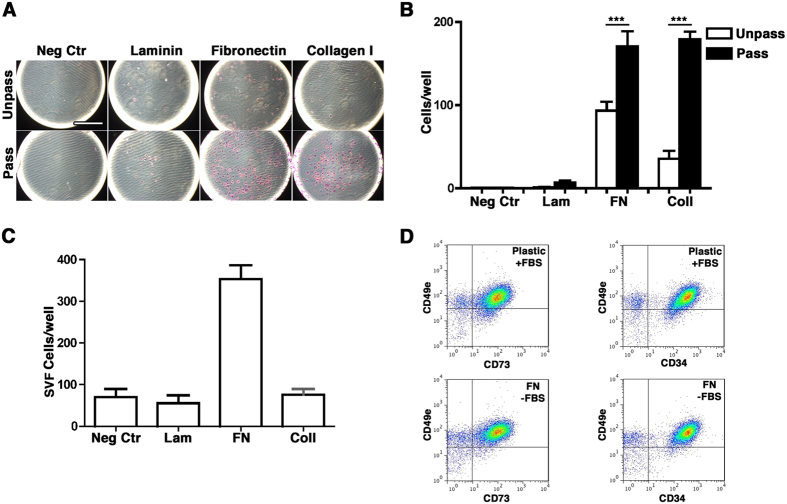
(**A**) Representative images of the adhesion assay performed with Unpass- and Pass-ASC in the absence of any coated protein (Neg Ctr), or on wells coated with either laminin (Lam), fibronectin (FN) or collagen type I (Coll). Size bar = 400 μm; (**B**) Quantification of the number of adhered cells per well in the absence of any protein (Neg Ctr), or on wells coated with either laminin (Lam), fibronectin (FN) or collagen type I (Col I) for Unpass- (white bar) and Pass-ASC (black bar). ***p < 0.001 (ANOVA with Bonferroni multiple comparisons test); (**C**) Quantification of the number of freshly isolated SVF cells adhered per well; (**D**) Representative cytofluorimetry plots of SVF cells stained for CD49e and CD73 (left panels) or CD49e and CD34 (right panels) after overnight seeding on plastic in presence of FBS (upper panels) or on plastic coated with fibronectin in absence of FBS (lower panels).

In agreement with the integrin expression profiles, adhesion assays performed in serum free conditions, demonstrated limited adhesion of Unpass-ASC to FN and collagen type I and no adhesion to laminin, whereas Pass-ASC showed significantly increased adhesion to both fibronectin (FN) and collagen type I (Coll) (Fig. 5A and B, n = 3, p < 0.001). Like Unpass-ASC, SVF cells adhered to FN but not to laminin (Fig. 5C). Next, and in order to investigate whether FN alone could mediate SVF cells adhesion, SVF cells were plated either on plastic tissue culture dishes in the presence of serum or on FN-coated dishes in the absence of serum. After overnight culture, adherent cells were harvested and characterized for the expression of CD34, CD73 and α_5_-integrin/CD49e. Populations similarly expressing the mesenchymal markers CD34, CD73 and CD49e were retrieved from either plastic or FN-coated dishes (Fig. 5D), indicating that FN alone could mediate SVF cells adhesion.

### Specific activation of α_5_-integrin (CD49e) decreases ASC bone forming potential *in vivo*

**Figure 6:**
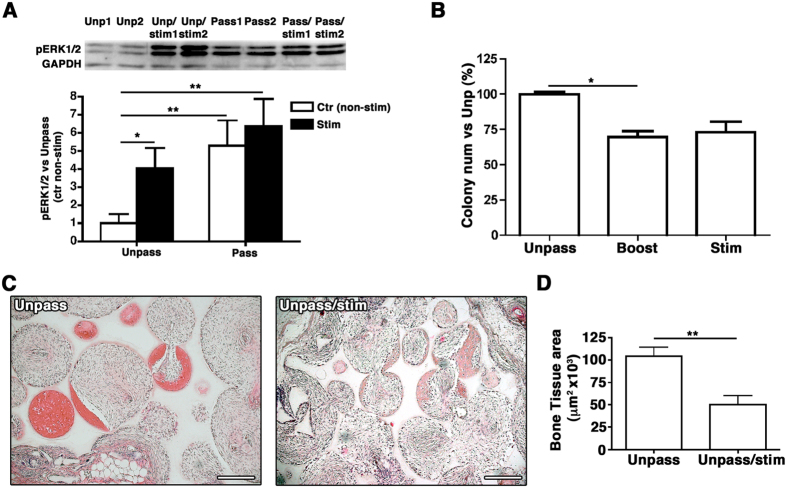
(**A**) Western Blot analysis of phospho -ERK1/2^thr202/tyr204^ in Unpass- and Pass-ASC without or with exposure to α_5_β_1_-integrin-specific peptide agonist CRRETAWAC (5 μg/ml, 30 minutes) stim condition. GAPDH was used as internal protein loading control. A representative blot following signal detection is shown. Values for levels of phospho -ERK1/2^thr202/tyr204^ were normalized with respect to their corresponding GAPDH value and are expressed relative to levels in unstimulated Unpass-ASC *p < 0.05, **p < 0.01 (ANOVA with Dunnet’s multiple comparisons test, after data normalization by logarithmic transformation). (**B**) Number of colonies (expressed as % of the colonies in the Unpass-ASC condition) of Unpass-ASC alone or after stimulation of the α_5_β_1_-integrin pathway only once (boost condition) or for the whole duration of the culture (stim condition). *P < 0.05 (Kruskal-Wallis with Dunn multiple comparisons test); (**C**) Representative fields of histological sections of osteogenic constructs loaded with Unpass-ASC or Unpass-ASC stimulated with CRRETAWAC (stim condition) and stained with H&E. Size bar = 200 μm; (**D**) Quantification of bone tissue formation 12 weeks after implantation of constructs loaded with Unpass-ASC or Unpass-ASC stimulated with the CRRETAWAC. **p < 0.01 (t-test).

We hypothesized that low expression of α_5_-integrin/CD49e would result in low activation of the downstream ERK1/2 pathway and maintain the osteogenic potential of Unpass-ASC and SVF cells. Indeed, Pass-ASC showed stronger ERK1/2 phosphorylation than Unpass-ASC (Fig. 6A, p < 0.01). Treatment of ASC with a specific α_5_β_1_-integrin-stimulating synthetic peptide^22^,^23^,^24^,^25^, CRRETAWAC, resulted in a 4-fold increase in ERK1/2 phosphorylation in Unpass-ASC (Fig. 6A, p < 0.05). Treatment of Unpass-ASC with the peptide only once (boost condition) significantly decreased the number of colonies (Fig. 6B, Unpass = 100 ± 1.9%, Boost = 69.6 ± 4.1% p < 0.05). The same trend was observed when the peptide was added for the whole duration of the culture (stimulation condition), although without statistical significance between the conditions (Stim = 73.2 ± 7.7% p = n.s., n = 3). Decrease of clonogenic potential was not accompanied by a change in expression of mesenchymal markers CD73 and CD90, which remained the same after treatment with the peptide (Unpass = 86.8 ± 7.6% and Stim = 93.5 ± 1.8% for CD73; Unpass = 93.8 ± 5.4% and Stim = 90.7 ± 9.8% for CD90). To determine whether activation of the ERK1/2 pathway through α_5_-integrin could also affect the osteogenic potential of Unpass-ASC, cells were stimulated or not with CRRETAWAC during *in vitro* culture on scaffolds in a perfusion bioreactor system. Following *in vivo* implantation of the scaffolds, ectopic bone formation was observed in both conditions (Fig. 6C). However, Unpass-ASC stimulated with CRRETAWAC gave rise to significantly lower amounts of bone tissue *in vivo* than untreated Unpass-ASC (Fig. 6D, n = 2, 5.0 ± 0.9 × 10^4^ μm^2^ with peptide *vs*. 10.4 ± 1.0 × 10^4^ μm^2^ without peptide, p < 0.05), supporting an association between a low level of ERK1/2 pathway activation through α_5_β_1_-integrin and the maintenance of ASC osteogenic potential.

### Alpha-5 integrin identifies a population of perivascular cells *in vivo* with clonogenic ability *in vitro*

**Figure 7:**
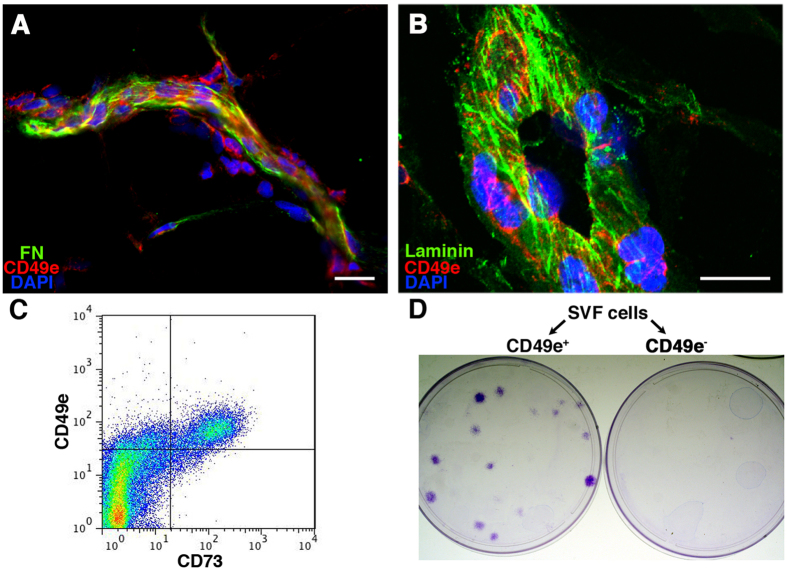
(**A**) Immunofluorescent staining of fibronectin (in green), α_5_-integrin/CD49e (in red) and nuclei (in blue) on frozen sections of human adipose tissue; (**B**) Immunofluorescent staining of laminin (in green), CD49e (in red) and nuclei (in blue) on frozen sections of human fat tissue. Size bar = 20 μm (**C**) Representative cytofluorimetry plot of SVF cells stained for CD73 and CD49e; (**D**) Representative images of colonies generated by the CD49e^+^ and CD49e^−^ sorted fractions of SVF cells.

To investigate which native cell population is identified by α_5_-integrin/CD49e, cryosections of human adipose tissue were stained for FN, α_5_-integrin/CD49e and laminin (Fig. 7). CD49e identified a population of cells in contact both with the vessel FN and the basal lamina (Fig. 7A and B, respectively). CD49e^+^ cells could be found only at a perivascular position, where the ASC niche was previously postulated to locate^26^. Furthermore, cytofluorimetric analysis showed that SVF cells contained a population of CD49e^Low^ cells also positive for the mesenchymal marker CD73 (Fig. 7C). Cell sorting experiments (n = 3) indicated that clonogenic progenitors were present only in the CD49e^+^/CD73^+^ fraction (Fig. 7D). However, only 15.7 ± 1.6% (n = 3) of the those cells gave rise to colonies when seeded onto tissue culture plastic, indicating that other cell types were included in this CD49e^+^/CD73^+^ population.

## Discussion

Key findings in this study are that long-term, non-passaged cultures of SVF cells generate a population of ASC with early progenitor features, and that FN receptor α_5_β_1_-integrin/CD49e plays a role in the maintenance of the *in vivo* osteogenic capacity by ASC. Unpass-ASC, as compared to passaged counterparts, displayed increased clonogenicity and bone tissue formation capacity comparable to that of freshly isolated, native SVF cells. This is in agreement with our previous report showing that even minimal expansion on plastic caused a significant loss of ASC osteogenic potential^3^. Unpass-ASC, despite the increased number of population doublings (Fig. 1C), generated a higher number of clonogenic cells and displayed a greater osteogenic potential *in vitro* and *in vivo* compared to P0-ASC. Our data show that SVF cells are able to generate an environment that preserves their bone tissue formation capacity. *In vivo*, the maintenance of stem/progenitor properties is tightly regulated by specialized microenvironments, called niches. Several studies have shown the importance of introducing specific niche signals during cell culture to preserve mesenchymal progenitors’ potential *in vitro*^10^,^15^. By providing both architectural elements and non-structural molecules to regulate the mechanical properties of the tissue and the differentiation of progenitor cells, the ECM is one of these essential niche components^17^. Culture of mesenchymal progenitors on decellularized ECM inhibits their spontaneous osteogenic differentiation and preserves their differentiation capacity^27^. During 28 days of culture, Unpass-ASC produced a layer of ECM containing FN but not collagen type I, and displayed adhesion to FN. FN has been recently described as a crucial component of the ECM for adhesion of muscle stem cells to their niche and a regulator of muscle stem cells maintenance and function^28^. Here, in adipose tissue we found perivascular cells in contact with vessel basal lamina FN, suggesting a possible role of this ECM protein in regulating also SVF cell biology.

Unlike the Unpass-ASC whose self-produced ECM remained intact for the entire 28 day duration of culture, Pass-ASC were treated multiple times with trypsin for cell detachment and passaging. By causing repetitive disruption of the ECM and of the cell-ECM interactions, this procedure might be detrimental to maintenance of ASC properties, as previously reported^29^. What remains unclear is whether the increased clonogenic and osteogenic functionality was due to maintenance of SVF progenitor properties during culture or to selection and expansion of a subpopulation of SVF cells with increased potency.

Our data show that in Unpass-, P0- and Pass-ASC conditions, cells homogenously expressed stromal markers and that contamination with mature hematopoietic and endothelial cells was minimal. However, Unpass-ASC significantly differed from P0 and Pass-ASC in their phenotype. In fact, even a short expansion on tissue culture plastic induced a change in the expression of several markers. Interestingly, CD105 was upregulated in P0 and Pass-ASC but remained low in both SVF cells and Unpass-ASC. Low expression of CD105 in Unpass-ASC should be further investigated since CD105 is downregulated in ASC with increased osteogenic potential through a reduction of transforming growth factor β1 (TGF-β1) signaling^30^. CD34, lost by P0 and Pass-ASC, was instead retained by a subpopulation of Unpass-ASC. We initially hypothesized that the increased differentiation potential of Unpass-ASC might be due to the presence of a CD34^+^ ASC population. CD34 has been described to be involved in the biology of several stem/progenitor cells, affecting adhesion, migration and proliferation^31^,^32^,^33^, and was also suggested as a marker of early progenitors in embryonic stem cell-derived MSC^34^. In SVF cells, the CD34^+^/CD31^−^ fraction contains all clonogenic and multipotent progenitors^35^. However, sorting experiments indicated that CD34^+^ cells displayed neither increased clonogenicity nor greater differentiation potential *in vitro* (Fig. 4). Moreover, CD34^−^ cells could re-express CD34 when plated and grown to confluence, suggesting CD34 might be modulated by culture conditions. Therefore, our data indicate that expression of CD34 is not responsible for the improved osteogenicity of ASC in this model and might rather have an anti-adhesive function under conditions of confluency, due to its negatively charged mucin domain, as previously described^33^.

Another major difference was the expression of integrins. Unlike P0- and Pass-ASC, Unpass-ASC maintained an integrin expression profile similar to that of SVF cells. Freshly isolated ASC did not express any of the main collagen receptors, such as α_1_-integrin (CD49a), that have been, instead, successfully used to isolate clonogenic populations from bone marrow samples^36^. Our data showed rather that Unpass-ASC and SVF cells expressed only low levels of the FN cell-surface receptor α_5_β_1_-integrin (CD49e/CD29), which undergoes upregulation during *in vitro* expansion on plastic. This is in line with previous reports showing that CD49e is involved in embryonic stem cell differentiation^37^,^38^ and upregulated during progenitor cell commitment^39^. CD49e also plays a role in the osteogenic differentiation of bone marrow-derived mesenchymal progenitors^40^ and in bone repair^41^. Furthermore, freshly isolated bone marrow-derived mesenchymal progenitors display a CD49e^low^ phenotype, and maintenance of a low expression of CD49e under appropriate culture conditions led to an increased osteogenic capacity^39^. Here, we showed that low expression of CD49e by ASC is similarly correlated with the maintenance of their osteogenic potential. In addition, activation of α_5_β_1_-integrin with a specific peptide activated the ERK1/2 pathway, decreased the number of clonogenic progenitors *in vitro* and reduced bone tissue formation *in vivo* by 50%. Several studies previously showed that the ERK1/2 pathway is involved in the proliferation and cell fate decision of progenitor cells^40^,^42^. Our data suggest support involvement of the ERK1/2 pathway in the osteogenic differentiation of ASC. In this study, activation of the ERK1/2 pathway was achieved through peptide-induced activation of α_5_β_1_-integrin. To confirm the role of α_5_β_1_-integrin, we overexpressed α_5_-integrin in Unpass-ASC using lentiviral vectors (data not shown). Although high expression of mRNA for α_5_-integrin was observed, the level of protein expression (assessed by immunostaining) was not different from non-transfected Unpass-ASC. This apparent discrepancy might be explained by the fact that α_5_-integrin is the only RGD-ligand subunit post-transcriptionally regulated by different miRNA^43^, suggesting a fine regulation of the expression levels of this integrin which might have occurred also in ASC. Future studies will thus aim at silencing α_5_-integrin during expansion of ASC in order to assess its impact on their *in vivo* bone forming capacity. Investigations on the role of other integrins in the maintenance of ASC progenitor properties are also warranted. Staining of human adipose tissue for α_5_-integrin identified a population of perivascular cells, and sorting of α_5_-integrin-positive SVF cells separated all clonogenic progenitors. SVF cells are highly heterogeneous and numerous markers have been proposed so far to specifically isolate clonogenic, osteogenic progenitors^44^,^45^,^46^. Our data show that α_5_-integrin, in combination with CD73, could be used to enrich for clonogenic progenitors but is not sufficiently specific for their selective isolation from SVF cells.

## Conclusions

We propose the establishment of a self-produced matrix containing FN for the *in vitro* expansion of SVF cells without an attendant loss of their *in vivo* osteogenic potential. The identification of the role of FN receptor α_5_β_1_-integrin, through ERK1/2 activation, in the maintenance of SVF bone forming capacity is an important step toward defining the required signals to fully preserve SVF native progenitor features. Further elucidation of other cellular and molecular components of the SVF native niche will advance efforts toward the engineering of a self-renewing environment for *in vitro* expansion of adipose-derived mesenchymal progenitors with full maintenance of their regenerative potency.

## Materials and Methods

### Cell isolation

Subcutaneous adipose tissue in the form of lipoaspirates was obtained from 20 healthy donors during routine liposuctions, after informed consent from the patient and following protocol approval by the ethical committee of the local Government (Permit number 78/07 of the Ethikkommission beider Basel, Kanton Basel-Stadt, Basel, Switzerland). All the methods were performed in accordance with the relevant guidelines and regulations. The tissue was digested in 0.075% collagenase type 2 (Worthington, Lakewood, NY) for 45 minutes at 37 °C on an orbital shaker. The suspension was thereafter centrifuged at 300 g for 10 minutes, and the resulting stromal vascular fraction (SVF) pellet was rinsed once with phosphate-buffered saline (PBS, Gibco, Grand Island, NY, USA), suspended in alpha-minimal essential medium (MEM) (Gibco), and filtered through a 100 μm strainer (BD Falcon; BD Biosciences, San Diego, CA, USA). Nucleated cells were counted after staining with 0.01% Crystal Violet (Sigma, St. Louis, MO, USA) in PBS.

### Cell culture

To determine colony forming efficiency, cells were plated at clonal density (9 cells/cm^2^) and cultured in alpha-MEM (Gibco) with 10% fetal bovine serum (FBS) and FGF-2 (5 ng/ml, R&D System, Minneapolis, MN, USA). After 2 weeks cultures were rinsed with PBS, fixed with 3.7% formaldehyde in PBS, stained with Crystal Violet for 10 minutes and rinsed with tap water. Colonies were counted. All determinations were performed in triplicate and colony-forming units-fibroblast (CFU-f) frequency in the fresh SVF sample was used to calculate the population doublings of first-confluence cultures.

For cell expansion, freshly isolated SVF cells were plated at a density of 1 × 10^5^ cells/cm^2^. When confluency was achieved, the cells were either detached with 0.05% trypsin/0.01% EDTA (Gibco) and re-plated at a density of 2 × 10^3^ cells/cm^2^ (Pass-ASC), or further cultured without passaging for 28 days (Unpass-ASC). Aliquots of the pooled cells were used for *in vitro* differentiation assays or for growth curve determination.

For the stimulation of α_5_ integrin^47^, a synthetic peptide (CRRETAWAC, Polypeptide, Strasbourg, France) was added to the culture medium at a concentration of 0.5 μg/ml.

### *In vitro* adipogenic differentiation

Adipogenic differentiation was induced in 2D cultures as previously described^15^. Briefly, cells were seeded in 6-well plates at a density of 5 × 10^3^ cells/cm^2^ and cultured in alpha-MEM with 10% FBS until they reached confluency. Medium was then supplemented with 10 μg/ml insulin, 10 μM dexamethasone, 100 μM indomethacin and 500 μM 3-isobutyl-1-methyl xanthine (adipogenic induction medium) for 72 hours and thereafter with 10 μg/ml insulin (adipogenic maintenance medium) for 24 hours. This 96-hours cycle was repeated four times, and then cells were cultured for an additional week in adipogenic maintenance medium. At the end of adipogenic induction, the cell monolayer was washed with PBS, fixed in 4% formalin for 10 minutes and stained with three volumes of Oil Red O (Sigma Aldrich AG, St. Louis, MO, USA) in 0.3% v/v isopropanol and two volumes of water for 15 minutes at room temperature.

Representative micrographs were acquired using a bright field microscope (Olympus IX50 with a Color View camera). Oil Red O, contained in lipid droplets, was then solubilized with 100% isopropanol and the optical density was measured with a spectrophotometer at 500 nm.

### *In vitro* osteogenic differentiation

Osteogenic differentiation was induced as previously described (9). Briefly, cells were seeded in 6-well plates at a density of 5 × 10^3^ cells/cm^2^ in alpha-MEM supplemented with 10% FBS, 10 mM β-glycerophosphate (Sigma), 10 nM dexamethasone (Sigma) and 0.1 mM L-ascorbic acid-2-phosphate (Sigma) and cultured for 3 weeks, with medium changes twice per week. Cells layers cultured in osteogenic medium were washed twice with PBS, fixed for 10 minutes in 4% formalin and washed twice with water. Fixed cells were incubated for 10 minutes with 2% alizarin red in water and thereafter extensively washed with water. Hydroxyapatite deposits were quantified using OsteoImage™ assay (Lonza, Basel, Switzerland, Catalog No. PA-1503), following manufacturer’s protocol. Briefly, cells were fixed in 70% ethanol for 20 minutes at room temperature, rinsed once with PBS and twice with the wash buffer provided. Cells were incubated with diluted staining solution at room temperature for 30 minutes under protection from light and then were rinsed with wash buffer. Mineralization was quantified using a plate reader at 492/520 nm (excitation/emission wavelengths). Data are reported as relative fluorescence units and expressed as mean ± SD.

### Real-time PCR

Total RNA was extracted from cells for each condition with RNeasy^®^ Mini kit protocol (Qiagen, Basel, Switzerland). All RNAs were treated by Deoxyribonuclease I (DNAse I; Invitrogen, Carlsbad, CA, USA) and total RNA was reverse-transcribed into cDNA with the Omniscript Reverse Transcription kit (Qiagen, Hombrechtikon, Switzerland) at 37 °C for 60 minutes. Quantitative real-time PCR assays were performed with ABIPrism 77000 Sequence Detection System (Perkin Elmer/Applied Biosystem, Rotkreuz, Switzerland) and utilizing Taqman Universal PCR Master Mix (Applied Biosystems, Forster City, CA, USA). The cycling parameters were: 50 °C for 2 minutes, followed by 95 °C for 10 minutes and 40 cycles of denaturation at 95 °C for 15 seconds and annealing/extension at 60 °C for 1 minute. Reactions were performed in triplicate for each template and specific gene expression was evaluated using the 2^ΔΔC^T method. Gene expression levels were normalized to the GAPDH mRNA as previously described^48^. Primers and probes for GAPDH (Hs02758991_g1), CD34 (Hs02576480_m1), Oct4 (Hs01895061_u1), Sox2 (Hs00602736_s1), Nanog (Hs02387400_g1) and Klf4 (Hs00358836_m1) were all provided by Assays-on-Demand, Gene Expression Products (Applied Biosystems, Foster City, California, United States).

### Assessment of bone formation *in vivo*

*In vivo* ectopic bone formation was assayed as previously described^3^. Briefly, hydroxyapatite scaffolds (Engipore, Finceramica-Faenza, Faenza, Italy) in the form of porous cylinders (0.8 cm diameter, 0.4 cm height) were placed into chambers of a previously developed perfusion-based bioreactor system^49^. Cells were suspended in CM supplemented with 10 nM dexamethasone, ascorbic acid (0.1 mM, Sigma), FGF-2 (5 ng/ml) and, when requested, the α_5_β_1_-stimulating peptide (0.5 μg/ml). The scaffolds were then perfused in alternate directions at a flow rate of 1 ml/min through the scaffold pores for 5 days, as previously described^3^ and then implanted subcutaneously in nude mice (Charles River, Wilmington, Massachusetts, United States,). Animals were treated in accordance with the Swiss Federal guidelines for animal welfare, after approval from the Veterinary Office of the Canton of Basel-Stadt (Basel, Switzerland). After 12 weeks, the constructs were harvested, fixed overnight in 4% formalin, completely decalcified with EDTA-based solution at 37 °C, paraffin embedded. 7 μm-thick-sections along the length of the construct were stained with haematoxylin and eosin (H&E) and Masson’s trichrome and then observed microscopically to detect the formation of bone tissue for qualitative analysis, and assessed by computerized bone histomorphometry as previously described^4^ for bone tissue quantification. Briefly, bright field images of sections at different depths of each construct were acquired and used to measure the area covered with bone tissue.

### Flow cytometry analysis and cell sorting

Cells (3–5 × 10^5^ cells) were suspended into 200 μl of 0.5% BSA in PBS (fluorescence-activated cell sorting [FACS] buffer) with conjugated antibodies against the indicated protein or an isotype control and were incubated for 30 minutes at 4 °C. The antibodies used were anti-human: CD49a-PE (559596), CD49b-FITC (555498), CD49c-PE (556025), CD49d-PE (555503), CD49e-PE (555617), CD29-PE (555443), CD41/61-FITC (555505), CD90-FITC (555595), CD73-PE (550257), CD34-APC (555824), CD14-FITC (555397), CD45-PE (555483), CD31-PE (555446), anti-mouse: IgG1-FITC (550616), IgG1-PE (554680), IgG2A-FITC (555573), IgG1-APC (all from Becton, Dickinson and Company, Franklin Lakes, NJ, www.bd.com), CD105-FITC (MCA1557F), CD49f-PE (MCA1457F) (both from Serotec Ltd., Oxford, U.K., www.serotec.com) and CD117-PE (130-091-734 from Miltenyi Biotech, Bergisch Gladbach, Germany). All antibodies were used at a dilution of 1:50, except CD105- FITC which was used at 1:20. Cells were washed twice with FACS buffer, suspended in PBS, and analyzed with a FACS- Calibur flow cytometer (Becton, Dickinson and Company, San Jose, CA, USA). For sorting experiments, freshly isolated SVF cells were stained for 30 minutes at 4 °C with antibodies specific for CD49e and CD73 or an isotype control, directly conjugated with PE or APC (BD Biosciences, Basel, Switzerland), in 0.5% BSA in PBS. The double positive population was then sorted with a FACS-Vantage SE cell sorter (Becton, Dickinson and Company, San Jose, CA, USA).

### Adhesion assay

Adhesion assays were performed as previously described^50^. Briefly, Microtiter plates (60 wells; Nunc) were coated for 1 hour at room temperature with 5 μl per well PBS containing 0.01% Tween and 40 μg/ml of Collagen Type I (354249, Becton, Dickinson and Company, San Jose, CA, USA), Laminin (from human fibroblasts, L4544, Sigma, Darmstadt, Germany) or Fibronectin (form human plasma, F0895, Sigma, Darmstadt, Germany). Each condition was tested in quadruplicate. All wells were then blocked for 30 minutes with PBS containing 1% BSA. Cells were plated (1.5 × 10^3^ cells/well), and after 1 hour the cells were fixed in 4% formaldehyde in PBS (30 minutes at room temperature) and stained with 0.1% crystal violet in water for 30 minutes. Cells were photographed with a Nikon microscope (Nikon Diaphot, Minato, Tokyo, Japan) equipped with a Nikon camera and counted.

### Immunohistochemistry and Immunofluorescence staining

After 28 days of culture, Unpass-ASC were fixed with PFA 1% for 1 hour and washed twice with PBS. Immunohistochemical staining using antibodies against Collagen type I (MP Biomedicals, Illkirch-Graffenstaden, France) and fibronectin was performed as previously described^8^. After incubation with a biotinylated secondary antibody and subsequently with an ABC-alkaline phosphatase complex, the specific staining was revealed by using Fast Red (All reagents from Dako, Baar, Switzerland). Matched IgG control antibodies were used as negative control.

For immunofluorescent staining, freshly harvested human fat tissue was frozen in liquid nitrogen, fixed in pure acetone for 10 minutes at −20 °C and then cryosectioned. Sections of 10 μm in thickness were stained with the following primary antibodies and dilutions: mouse anti-human CD49e (BD Biosciences, Basel, Switzerland) at 1:50; mouse anti-human; rabbit monoclonal anti-fibronectin (Abcam, Cambridge, UK) at 1:200; and rabbit polyclonal anti-laminin (Abcam) at 1:50. Fluorescent-conjugated secondary antibodies (Invitrogen, Basel, Switzerland) were used at 1:200. Images were acquired with a Nikon A1R confocal microscope.

### Western blot

Cells were rinsed with PBS and lysed in lysis buffer containing 25 mM Tris-HCl (pH 7.4), 150 mM NaCl, 1% NP-40, 1 mM EDTA, 5% glycerol, phosphatase inhibitor cocktail and protease inhibitor cocktail (both from Roche Diagnostics (Schweiz) AG, Risch-Rotkreuz, Switzerland). Protein concentrations were determined using the BCA Protein Assay Kit (ThermoScientific (Schweiz) AG, Reinach, Switzerland). Crude cell protein lysates (10 μg/lane) were subjected to standard SDS-polyacrylamide gel electrophoresis under reducing conditions and electro-blotted onto nitrocellulose. Membranes were immuno-probed using primary antibodies against phospho -ERK1/2^thr202/tyr204^ (Cell Signaling Technology) and GAPDH (Abcam, UK) as the internal protein loading control. Secondary HRP-conjugated anti-species specific IgGs were from Southern Biotechnology (BioReba AG, Reinach, Switzerland). Immunoreactive proteins were detected using Pierce ECL Western blotting substrate (Thermo Scientific) with signal capture using the Bio-Rad Molecular Imager Gel Doc XR+ system (Bio-Rad Laboratories, Hercules, CA, USA) are shown. Signal intensities were quantified using Image J software (https://imagej.nih.gov/ij/) and to correct for variations in sample loading pERK1/2 values were normalized with respect to their corresponding GAPDH value.

### Statistical analysis

Results are expressed as mean ± SEM. Before statistical testing, Kolmogorov-Smirnov test was performed on all data sets to assess normal distribution. When data did not satisfy the normality test, they were analyzed with the non-parametric Kruskal-Wallis test for multiple comparisons and Dunn’s post-hoc test or with Mann-Whitney test for single comparison. Data sets that passed the normality test were analyzed with 1-way ANOVA with Bonferroni’s or Dunnet’s post-test for multiple comparisons or with t test for single comparison. Results were considered to be statistically significant at p values < 0.05 (*p < 0.05, **p < 0.01, ***p < 0.001). The data were processed with GraphPad Prism 5 Software (GraphPad; San Diego, CA, USA).

## Additional Information

**How to cite this article**: Di Maggio, N. *et al*. Extracellular matrix and α_5_β_1_ integrin signaling control the maintenance of bone formation capacity by human adipose-derived stromal cells. *Sci. Rep.* **7**, 44398; doi: 10.1038/srep44398 (2017).

**Publisher's note:** Springer Nature remains neutral with regard to jurisdictional claims in published maps and institutional affiliations.

## Supplementary Material

Supplementary Information

## Figures and Tables

**Table 1:** Analysis of surface marker expression in freshly isolated SVF cells, P0-ASC, Unpass-ASC and Pass-ASC (N.D. = not detectable).

	SVF	P0-ASC	Unpass-ASC	Pass-ASC
**CD34**	41.1 ± 0.2	24.3 ± 16.3	35.3 ± 14.7	2.4 ± 2.7
**CD29**	57.7 ± 10.5	100.0 ± 0.1	52.2 ± 18.8	99.7 ± 0.2
**CD49a**	8.1 ± 2.9	35.8 ± 7.4	5.5 ± 5.2	70.3 ± 6.2
**CD49b**	1.4 ± 0.7	96.0 ± 3.4	5.2 ± 1.9	98.8 ± 1.1
**CD49c**	2.5 ± 0.3	90.5 ± 7.8	2.7 ± 0.9	81.1 ± 23.5
**CD49d**	13.1 ± 8.4	98.8 ± 1.4	1.7 ± 0.9	95.5 ± 7.1
**CD49e**	61.1 ± 2.7 (low)	100.0 ± 0.1(high)	96.4 ± 3.2 (low)	99.9 ± 0.1(high)
**CD49f**	16.2 ± 0.2	1.3 ± 0.7	1.5 ± 0.6	0.9 ± 0.7
**CD51/61**	1.3 ± 1.2	86.7 ± 13.6	20.3 ± 1.4	92.8 ± 1.6
**CD105**	9.8 ± 4.7	100.0 ± 0.1	6.3 ± 6.9	99.7 ± 0.2
**CD73**	57.0 ± 10.8	99.6 ± 0.5	93.9 ± 5.2	99.2 ± 1.3
**CD90**	56.6 ± 12.3	99.8 ± 0.1	98.3 ± 0.6	99.3 ± 0.6
**CD45**	21.6 ± 14.2	0.4 ± 0.8	0.3 ± 0.3	N.D.
**CD14**	22.2 ± 8.8	1.0 ± 0.8	0.3 ± 0.2	N.D.
**CD31**	27.0 ± 12.1	0.7 ± 0.8	0.2 ± 0.2	N.D.
