# Supplementary material for: Extracellular matrix and α5β1 integrin signaling control the maintenance of bone formation capacity by human adipose-derived stromal cells
Source: Sci Rep. 2017 Mar 14;7:44398. doi: 10.1038/srep44398 (PMC5349595; doi:10.1038/srep44398)
Supplement: Supplementary Information [file srep44398-s1.pdf]

Supplementary Information

Title

Extracellular matrix and $\alpha_5\beta_1$ integrin signaling control the maintenance of bone formation capacity by human adipose-derived stromal cells

Authors

Nunzia Di Maggio, Elisa Martella, Agne Frismantiene, Therese J Resink, Simone Schreiner, Enrico Lucarelli, Claude Jaquiery, Dirk J Schaefer, Ivan Martin and Arnaud Scherberich

Supplementary Figure 1

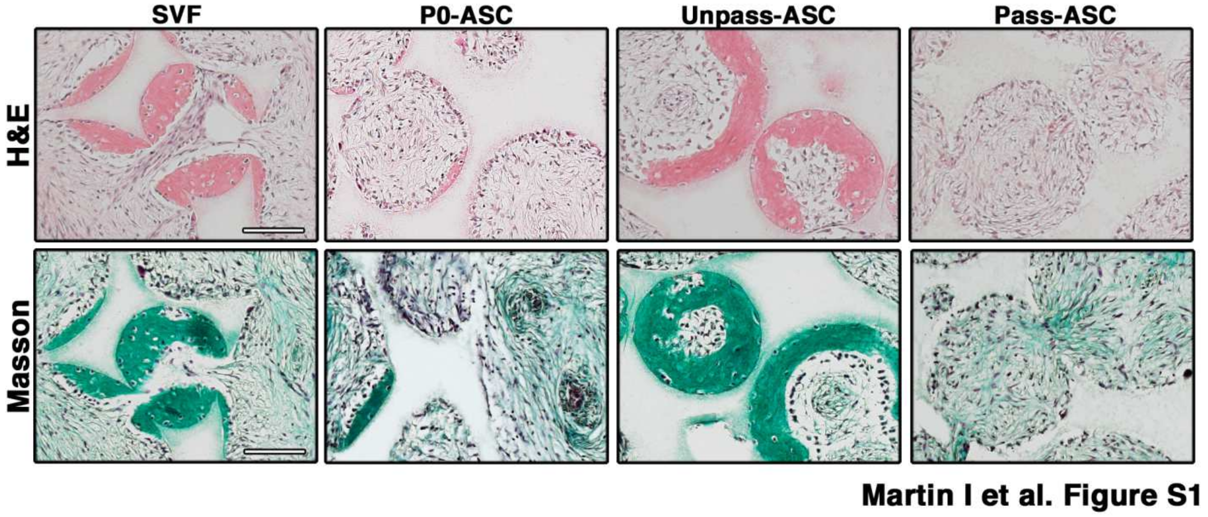

Figure S1. Representative microscopy fields of histological sections of constructs loaded with SVF cells, P0, Unpass- and Pass-ASC, stained with H&E and Masson's trichrome staining. Size bar = 100 µm.
